# Knowledge domain and emerging trends in post-stroke cognitive impairment: a bibliometric analysis

**DOI:** 10.3389/fnagi.2025.1525626

**Published:** 2025-03-04

**Authors:** Heyu Zhang, Yanwei Li, Luqian Zhan, Jingfang Long, Jianing Shen, Jiahui Chen, Jiajing Qian, Zhiming Pan, Xue Wu, Zhen Wang, Wenjun Wu, Guiqian Huang

**Affiliations:** ^1^Department of Neurology, The First Affiliated Hospital of Wenzhou Medical University, Affiliated With Wenzhou Medical University, Wenzhou, China; ^2^Department of Neurology, Wenzhou Hospital of Integrated Traditional Chinese and Western Medicine, Wenzhou, China; ^3^Department of Neurology, Wenzhou Central Hospital, Wenzhou, China; ^4^Department of Mental Health, The First Affiliated Hospital of Wenzhou Medical University, Affiliated With Wenzhou Medical University, Wenzhou, China; ^5^Department of Thyroid and Breast Surgery, The First Affiliated Hospital of Wenzhou Medical University, Affiliated With Wenzhou Medical University, Wenzhou, China; ^6^Key Laboratory of Alzheimer's Disease of Zhejiang Province, Institute of Aging, Wenzhou, China; ^7^Department of Endocrinology, The First Affiliated Hospital of Wenzhou Medical University, Wenzhou, Affiliated With Wenzhou Medical University, Wenzhou, China; ^8^School of Mental Health, Affiliated With Wenzhou Medical University, Wenzhou, China

**Keywords:** stroke, cognitive impairment, post-stroke cognitive impairment, cerebral small vessel disease, bibliometrics, visual analytics, VOSviewer, CiteSpace

## Abstract

**Background:**

Cognitive impairment is an important cause of disability and death among the elderly. One of the most important risk factors is stroke. Post-stroke cognitive impairment (PSCI) not only diminishes the quality of life for patients but also increases the burden on families and society. But PSCI can be mitigated through early intervention. Cerebral small vessel disease (CSVD) is one of the significant causes of stroke and has garnered considerable attention in PSCI. Therefore, this study aims to identify research priorities and trends in PSCI through bibliometric analysis, and further explore the role played by CSVD in PSCI.

**Methods:**

In this study, we performed a systematic search in the Science Citation Index Expanded (SCI-E) of the Web of Science Core Collection (WoSCC). VOSviewer, CiteSpace and Origin were mainly used to visualize the research focus and trend in PSCI. In addition, we screened the retrieved literature again, and performed keyword analysis on the studies related to CSVD.

**Results:**

A total of 1,943 publications were retrieved in the field of PSCI in this study, with consistent upward trend in annual publications in recent years. Pendlebury was an important leader in PSCI research. Capital Medical University was in the leading position judging from the number of publications. China had the highest number of publications in this field. The journal Stroke had the strongest international influence in this field. Keywords such as “functional connectivity,” “tool,” “systematic review,” and “meta-analysis” have been revealed to have momentous impact on PSCI in recent years. In the further analysis of PSCI and CSVD, “hypertension,” “white matter hyperintensities (WMH),” “cerebral microbleeds (CMBs),” and “cerebral amyloid angiopathy (CAA)” received extensive attention.

**Conclusion:**

The study of PSCI is still in the development stage. This study systematically summarizes the progress and development trend in the field of PSCI, and further explores the relationship between CSVD and PSCI through hypertension and magnetic resonance imaging markers. This study is of great significance for researchers to quickly understand the development of PSCI, but also helps them understand future directions, and provides important insights for the prevention and treatment of PSCI.

## 1 Introduction

Cognitive functions include memory, learning, comprehension, orientation, judgment, calculation, language, visuospatial skills, analysis, and problem-solving. Cognitive impairment refers to varying degrees of cognitive dysfunction due to various causes ranging from mild to severe and manifests as subjective cognitive decline (SCD) (Jessen et al., 2020), mild cognitive impairment (MCI), or dementia (Sachdev et al., 2009; Knopman and Petersen, 2014). Cognitive impairment is a major cause of disability and death in the elderly (Pike et al., 2022). With an aging social population, the number of individuals suffering from cognitive impairment is increasing, placing a heavy burden on families and society (Jia et al., 2018). Thus, improving cognitive impairment is still the focus of current research.

Vascular cognitive impairment (VCI) caused by cerebrovascular diseases, such as stroke, is the second most common type of dementia after Alzheimer's disease (AD) (Wolters and Ikram, 2019). Stroke is an important risk factor for cognitive impairment, and the location, size, severity (Pendlebury and Rothwell, 2009; Rost et al., 2022), and recurrence frequency of cerebral infarction (Pendlebury and Rothwell, 2019) are associated with post-stroke dementia (PSD). Post-stroke cognitive impairment (PSCI) is a clinical syndrome characterized by cognitive deficits that persist for 6 months after a stroke. Cognitive impairment after stroke seriously affects patients' quality of life and survival time, and increases the risk of disability and death (El Husseini et al., 2023).

Most cognitive impairments (especially neurodegenerative cognitive disorders) are insidious in onset, slow in progression, and characterized by progressive worsening (Chinese Expert Consensus Committee on Brain Cognitive Health Management Chinese Journal of Health Management Editorial Committee, 2023). However, cognitive function decline in patients with PSCI often has a delayed onset, during which intervention measures can be implemented to improve cognitive function in stroke survivors (Brainin et al., 2015a). However, even in patients who initially recover from cognitive impairment post-stroke, there is an increased risk of progression to dementia (El Husseini et al., 2023). Therefore, early detection and prevention of PSCI are critical. PSCI has become a popular topic in international stroke research and intervention (Dong et al., 2017).

Lacunar cerebral infarction caused by CSVD accounts for 25–50% of ischemic stroke (Tsai et al., 2013; Georgakis et al., 2023). Furthermore, CSVD is the leading cause of VCI, accounting for 36–67% of vascular dementia (VaD) (Luo et al., 2021), which has a significant impact on society. CSVD is easily ignored; however, with the development of MRI, the diagnostic rate for CSVD has significantly improved. An increasing number of studies have established the central role of CSVD in cognitive impairment (Geriatric Neurology Group of Chinese Society of Geriatrics Clinical Practice Guideline for Cognitive Impairment of Cerebral Small Vessel Disease Writing Group, 2019). Consequently, CSVD has attracted significant attention in the context of PSCI. Therefore, it is necessary to analyze the research status, development trends, and frontier hotspots of PSCI- and CSVD-related fields.

Bibliometrics can comprehensively display the research content of a certain field and predict new trends through statistical and quantitative analyses of scientific data within publications (Lin et al., 2022; Wei et al., 2022). This research method has been widely used in many fields including cancer (Zhang et al., 2020), cardiovascular diseases (Bloomfield et al., 2015), respiratory diseases (Lin et al., 2022), nervous system diseases (Liu et al., 2019; Quispe-Vicuña et al., 2022), and so on. However, although bibliometrics have made some progress in the field of PSCI (Chi et al., 2023; Ou et al., 2023), the association between PSCI and CSVD has not yet been studied. Therefore, the purpose of this paper was to systematically analyze the research in the field of PCSI, further explore the role of CSVD in PSCI, provide a scientific basis for clinical practice, promote early diagnosis and treatment of PSCI, and encourage innovative research in the prevention and treatment of PSCI. At the same time, we sought to identify future research hotspots through bibliometrics, guiding researchers and decision-makers in adjusting future research directions and strategies to promote rapid and efficient development in this field, ultimately improving the quality of life for patients and their families. Additionally, through bibliometrics, we aimed to help junior researchers identify mentors and partners, select appropriate journals, and evaluate corresponding institutions, thereby expanding cooperation and obtaining more accurate academic support and resources.

## 2 Methods

### 2.1 Data sources and search strategy

**Figure 1:**
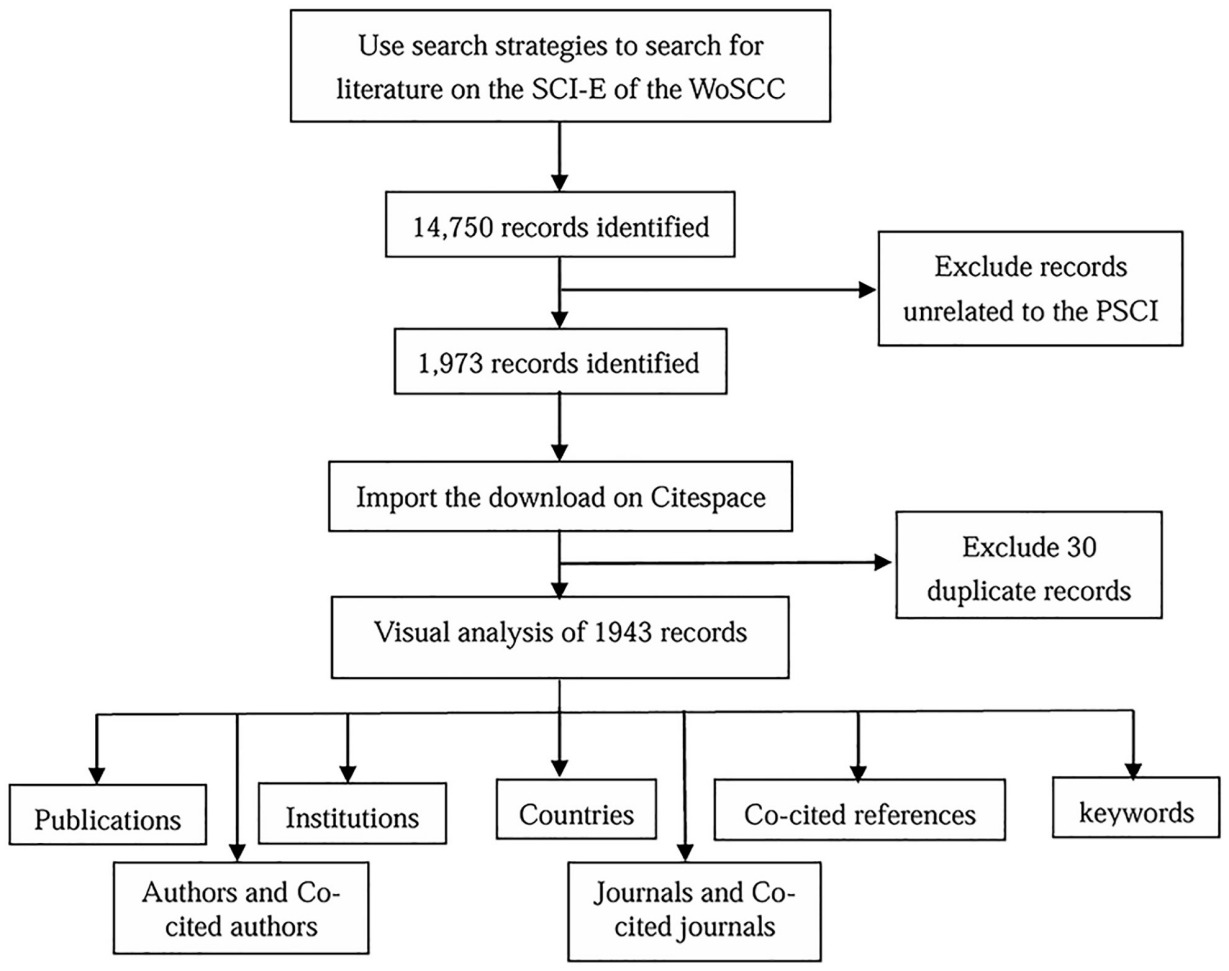
The workflow of this study.

The Web of Science platform is considered one of the most authoritative citation index databases in the world (Wei et al., 2022; Meng et al., 2024). We conducted a systematic search in the SCI-E of the WoSCC database on February 6, 2023. The following retrieval strategy was employed: TS=((Stroke AND “Cognitive dysfunction”) OR “PSCI”). The specific search strategies are recorded in Supplementary material. The literature published between January 1, 2010, and January 31, 2023, was included. After excluding articles written in non-English, we obtained 14,750 original records. After removing the articles not relevant to PSCI and repeated, we finally obtained 1,943 records (Figure 1). Notably, 1,713 research papers were published, while the remaining types of literature included reviews (*n* = 182), early access (*n* = 34), meeting abstract (*n* = 8), and proceedings paper (*n* = 6).

### 2.2 Data analysis

Extracting the original data from the SCI-E database of the WOSCC inevitably leads to duplicate entries. Therefore, we cleaned the author, country, keywords and other information, and finally imported the cleansed data to VOSviewer (version 1.6.19; Leiden University, Netherlands) and CiteSpace (version 6.2. R3; Drexel University, PA, United States) software applications (van Eck and Waltman, 2010; Chen, 2004) for the subsequent bibliometric analysis. The specific removal process is shown in Supplementary material. Additionally, we utilized Tableau Public software to display the geographical distribution of publications across different countries. Furthermore, we collaborated with the Microsoft Charticulator website^1^ to create a string map illustrating international collaboration between clusters. Ultimately, we imported the co-cited references and keyword data analyzed by CiteSpace (version 5.7. R5) into Mapequation^2^ to generate an alluvial flow graph.

## 3 Results

This study analyzed 1,943 publications from 76 countries, involving 2,356 institutions and 9,709 authors, published across 465 journals and citing 53,566 references from 7,233 different journals.

### 3.1 Annual trends in publication volume

**Figure 2:**
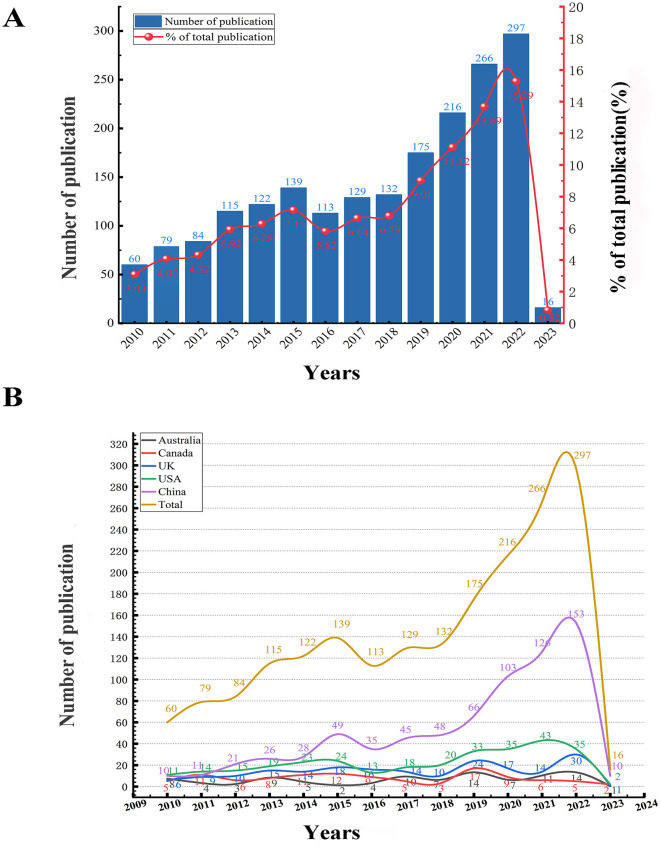
**(A)** The annual trend of paper publication quantity in the PSCI field. The literature published between January 1, 2010, and January 31, 2023, was included. **(B)** Annual trends in the number of publications in all countries and the top five countries in the PSCI field.

Analyzing the annual publication volume provides insights into the evolution of research focus and trends within a specific field. In this study, we finally obtained 1,943 records. Since this study commenced in February 2023, we included publications up to January 2023. As shown in Figure 2A, the annual number of papers shows an upward trend, with the publication volume in 2022 being 4.95 times that of 2010, indicating a growing academic interest in PSCI research. Excluding 2023, the publication trend can be divided into two phases: the first 6 years experienced stable growth, with 599 papers published, while the following 7 years saw a faster increase, with 1,328 papers published. This upward trend suggests a strong and continuing research interest in PSCI, highlighting the field's significant potential for exploration.

We analyzed the number of publications from all countries and highlighted the top five countries during this period (Figure 2B). We found that the annual publication trend in China closely mirrored the global trend, with China consistently leading in publication volume each year. Following China, the USA, the UK, Canada, and Australia also made significant contributions to PSCI research. Among these, the USA exhibited the highest annual publication quantity, although the difference in publication volume between the USA and the other three countries was not substantial. In conclusion, other countries except China may not yet have entered an explosive growth period.

### 3.2 Analysis of authors and co-cited authors

**Figure 3:**
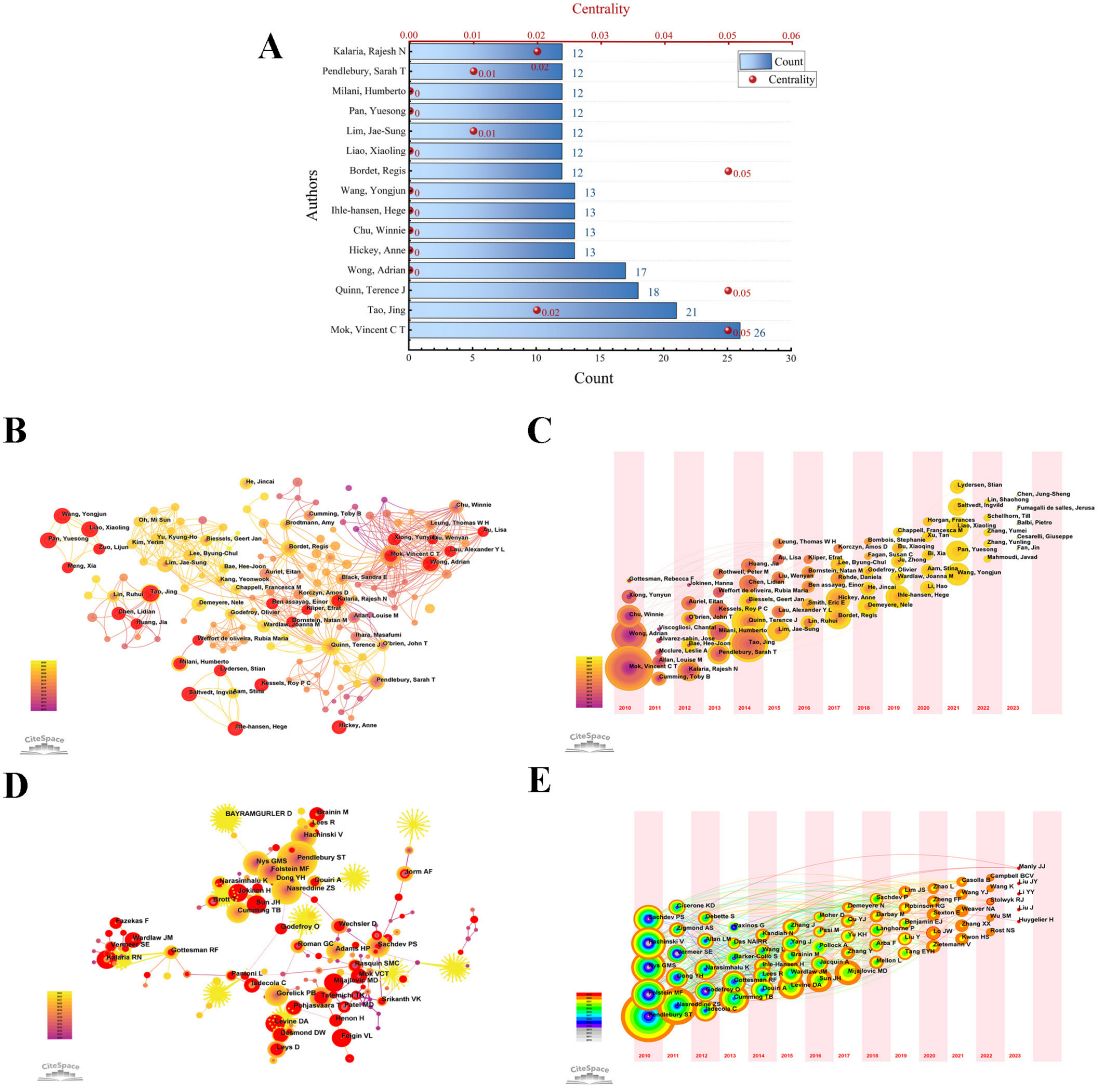
Authors related to the research in PSCI. **(A)** The publication quantity and centrality of the top 15 authors in the PSCI field. **(B)** The author's visualization map. The larger the node is, the higher the author 's publication is. The time of publication is reflected by the depth of color. **(C)** The author 's timezone map related to PSCI. Different annual rings represent different authors; and the time when the node appears is the time when the author first published the article during the study period; the color reflects the corresponding time. With the change of time, the accumulation of publications is expressed by the size of the annual ring. If the author participates in the same article as the previous author, there is a connection between them, but there is no connection between authors who appear together in the same year. **(D)** Network of co-cited authors based on CiteSpace. When two authors are cited together in the same article, a co-citation relationship is established. Notably, centrality values **>** 0.1 would be indicated by a purple circle. Nodes with higher centrality may be positioned in the center of large clusters or subnetworks, indicating the interdisciplinary potential of the node and suggesting that the author's research content is multidisciplinary. **(E)** The timezone map of co-cited authors.

Through VOSviewer visualization analysis, we found that a total of 9,709 authors contributed to this field during the study period. Additionally, we analyzed the authors' collaboration network using CiteSpace software, yielding important metrics such as publication volume and centrality, which are depicted in a bar chart generated by Origin (Figure 3A). The bar chart shows that Vincent C. T. Mok has the most articles (*n* = 26), followed by Jing Tao (*n* = 21), Terence J. Quinn (*n* = 18), and Adrian Wong (*n* = 17). Among these, Mok, Quinn, and Bordet had the highest centrality (0.05), although these values were relatively low.

Figure 3B presents the author collaboration map based on CiteSpace, which helps identify core authors and the intensity of collaboration between them. The lines between nodes stand for authors' collaboration. The author timezone map (Figure 3C) illustrates the distribution and changes in authors' contributions over different periods. Among the top 15 authors, the earliest contributors were Mok, Wong, and Chu, who laid the groundwork for PSCI research, while Wang, Xiaoling Liao, and Yuesong Pan began publishing in this field after 2021.

Figure 3D displays the co-cited author network. After adjusting the network, we obtained 752 nodes and 2,338 links. As shown in Figure 3D and Table 1, the top five co-cited authors are Pendlebury (*n* = 525, 0), Nasreddine (*n* = 300, 0.04), Folstein (*n* = 249, 0.03), Nys Gms (*n* = 234, 0), and Hachinski (*n* = 220, 0.01). Notably, Pendlebury had the highest number of publications but a centrality of 0, suggesting that this author's research content is relatively specialized in PSCI. In contrast, Cordonnier had the highest centrality score at 0.31, despite contributing a comparatively lower number of publications (31).

**Table 1:** The top 10 co-cited authors with the highest number of publications and centrality in the PSCI field respectively.

**Rank**	**Co-cited author**	**Year**	**Count**	**Centrality**	**Co-cited author**	**Year**	**Count**	**Centrality**
1	Pendlebury ST	2010	525	0	Cordonnier C	2011	31	0.31
2	Nasreddine ZS	2011	300	0.04	Barker-Collo Sl	2010	22	0.3
3	Folstein MF	2010	249	0.03	Allan Lm	2012	54	0.26
4	Nys Gms	2010	234	0	Hochstenbach J	2010	23	0.24
5	Hachinski V	2010	220	0.01	Alawieh A	2023	1	0.24
6	Sachdev PS	2010	192	0.01	Li Y	2016	19	0.23
7	Gorelick PB	2010	185	0.01	Kirino T	2011	32	0.18
8	Tatemichi TK	2010	185	0	Ballard C	2010	81	0.17
9	Cumming TB	2013	174	0.04	Tang Wk	2010	64	0.17
10	Jokinen H	2010	148	0.08	Fazekas F	2010	92	0.16

We adjusted the network to visualize the co-cited authors with a minimum of five citations for time-zone graph analysis (Figure 3E), resulting in 880 nodes and 2,150 links. Pendlebury's node demonstrated the widest yellow ring, suggesting that the literature published by this author received significant attention from scholars in PSCI in 2021. Similarly, Nasreddine's node exhibited the widest orange annual ring, indicating that his publications in PSCI in 2022 were highly cited by many researchers. The differing focus of various scholars over time highlights the evolution of research trends in this field.

### 3.3 Institutional analysis

**Figure 4:**
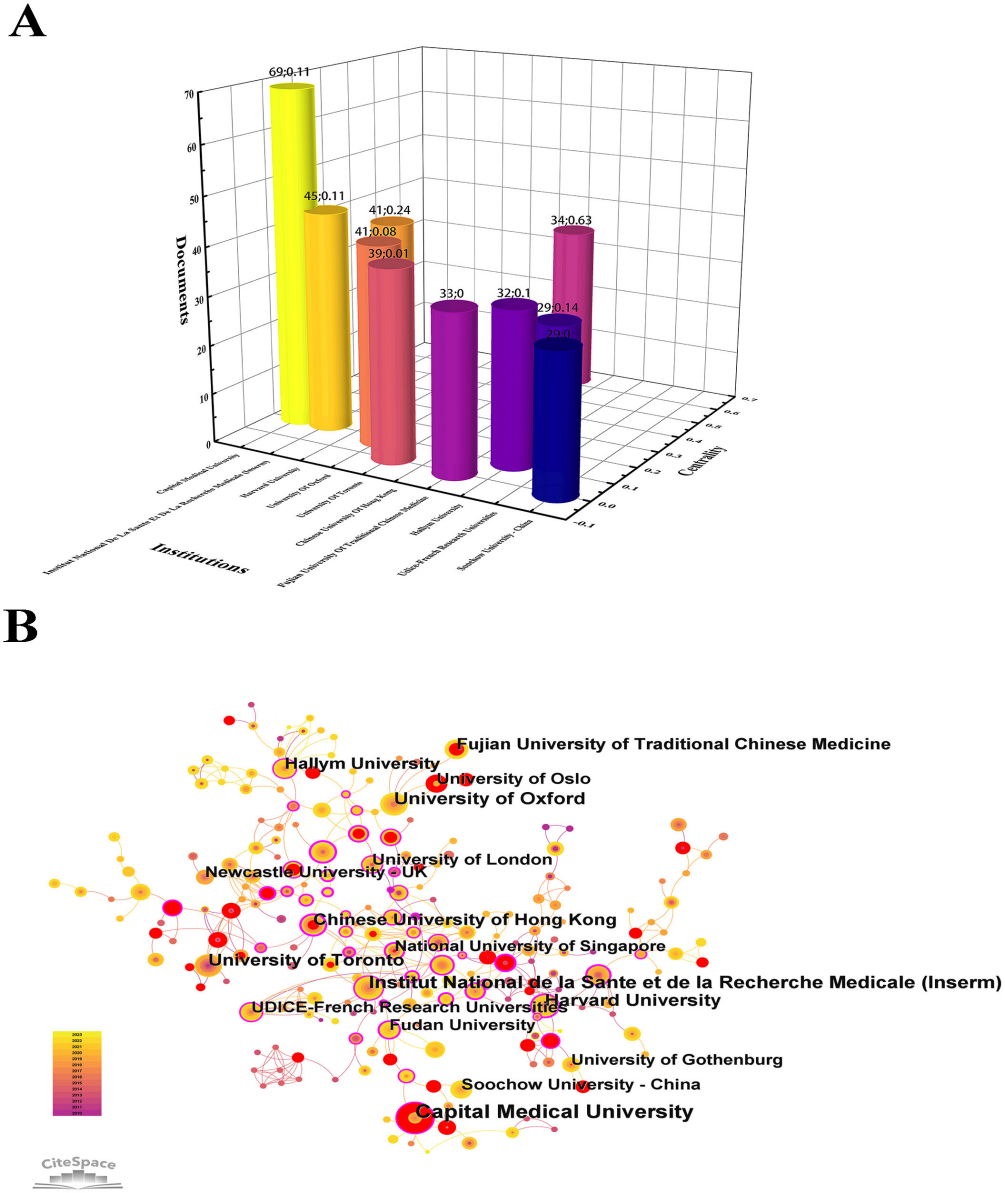
Visualization of institutions in PSCI. **(A)** Top 15 institutions engaged in PSCI and their centrality. Different color histograms represent different institutions. The number of institutional publications is related to the height of the histogram. The distance between the bar chart and the *X*-axis reflects the centrality of the institution. **(B)** Network of institutions engaged in PSCI. Each node represents an institution, and a larger node represents more publications. If the average time of publishing PSCI articles is later, the node color is lighter. Nodes with red rings represent institutions with a sudden increase in the number of publications over a period of time. The purple circle indicates that the centrality of the institution is >0.1.

Within the PSCI field, 2,356 institutions played various roles during the study period. We used Origin software to create a three-dimensional histogram of institutional information (Figure 4A) and CiteSpace to construct an institutional visualization map (Figure 4B). With a g-index of 15, the network included 267 nodes and 1,026 connections. Combined with Table 2 and pictures, we find that the institution with the highest number of publications was Capital Medical University (*n* = 69), followed by Institut National De La Sante Et De La Recherche (*n* = 45), Harvard University (*n* = 41), University of Oxford (*n* = 41), and University of Toronto (*n* = 39). Taken together, although the Chinese University of Hong Kong did not have the highest number of publications (*n* = 34), it had the highest centrality ranking among all institutions (0.63), indicating a strong influence in this field. The next institutions with high centrality were the University of Glasgow (0.49). Overall, most institutions are closely connected and collaborative.

**Table 2:** The top 10 institutions with the highest number of PSCI publications and centrality, respectively.

**Rank**	**Institutions**	**Documents**	**Centrality**	**Institutions**	**Documents**	**Centrality**
1	Capital Medical University	69	0.11	Chinese University of Hong Kong	34	0.63
2	Institut National De La Sante Et De La Recherche	45	0.11	University of Glasgow	24	0.49
3	Harvard University	41	0.24	Shandong First Medical University & Shandong	3	0.46
4	University of Oxford	41	0.08	Fudan University	28	0.39
5	University of Toronto	39	0.01	University College London	5	0.37
6	Chinese University of Hong Kong	34	0.63	Karolinska Institutet	7	0.34
7	Fujian University of Traditional Chinese Medicine	33	0	Harvard University	41	0.24
8	Hallym University	32	0.1	CHU Lille	22	0.23
9	Udice-French Research Universities	29	0.14	US Department of Veterans Affairs	18	0.23
10	Soochow University—China	29	0	National University of Singapore	27	0.22

### 3.4 Country analysis

**Figure 5:**
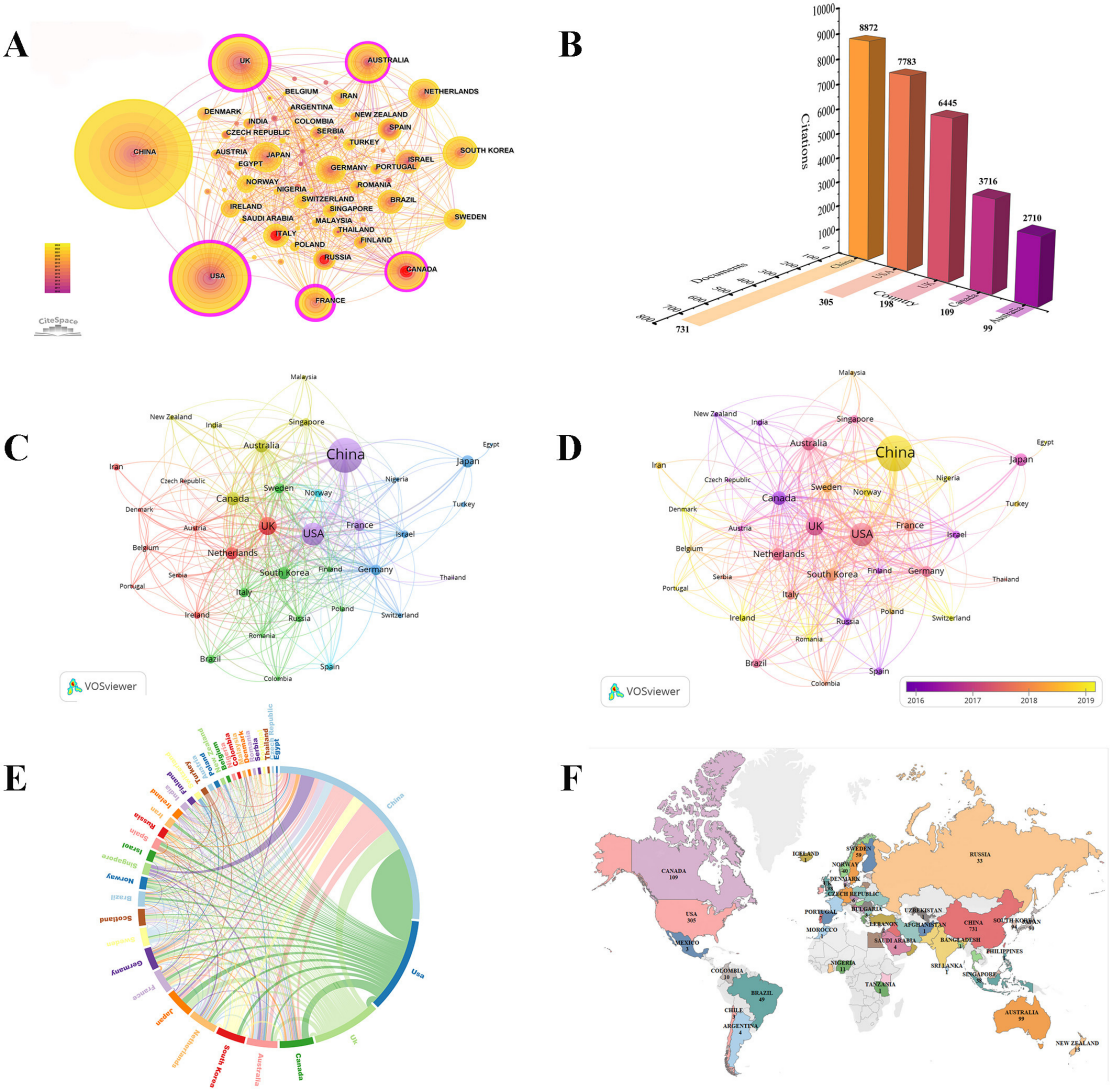
Bibliometric analysis of countries in the PSCI field. **(A)** A visual map for CiteSpace network among countries. **(B)** Shadow image. The *Y*-axis numbers and *Z*-axis numbers respectively indicate the number of PSCI documents and citations in the top five countries. **(C)** Collaboration network of countries based on VOSviewer. **(D)** Overlay visualization map of countries analysis. **(E)** A circle diagram evaluates the international collaboration between clusters. A circle diagram evaluates the international collaboration between clusters. The country is arranged in a circle in the form of an arc, and different countries are distinguished by different colors. The arc becomes shorter in the clockwise direction, indicating that the number of national publications is decreasing. The thicker the connection between countries, the stronger the degree of cooperation between the two. **(F)** National geographic distribution map.

During the study period, researchers from 76 countries contributed to the PSCI field. Notably, China had the highest publication output (*n* = 731, 37.6%) and total citations (*n* = 8,872); however, the country's average number of citations (12.14) and centrality (0.01) were relatively low. The countries with the next highest publication yields were the USA (*n* = 305, 15.7%), the UK (*n* = 198, 10.2%), Canada (*n* = 109, 5.6%), and Australia (*n* = 99, 5.1%) (Supplementary Table S1; Figures 5A, B). Among these, Canada had the highest average number of citations (34.09), while the UK had the highest centrality (0.28). Canada has garnered significant attention from scholars, and the UK acts as a vital bridge across various PSCI domains. Upon careful observation of Figure 5A, we see that the color width for China in 2022 is notably wide. Combined with Figure 2B, this indicates that China is highly engaged in PSCI research, suggesting that PSCI has become a key focus in Chinese neurology. It is important to note that for countries such as Canada, Italy, and Russia, the red rings indicate periods of heightened publication activity, drawing significant attention from researchers.

Furthermore, we used VOSviewer software to create Figures 5C, D, which represent the network view and overlay view of international collaboration in PSCI research, respectively. Regarding cooperation between countries, we used the Microsoft Charticulator website to create a chord diagram (Figure 5E), allowing us to observe that the thickest cyan arc lies between China and the USA. This relationship indicates that both countries dominate publication output and have the most frequent cooperative exchanges. Notably, the UK had the most connections, suggesting it has the most collaborative partners. Additionally, among the top five countries, China had the least international cooperation, indicating that China should engage in broader international collaborations to explore new areas within PSCI research (Supplementary Table S1; Figures 5C, E).

Using VOSviewer, we constructed a map color-coded to represent the average year of publication by country (Figure 5D). China, Denmark, Portugal, Lreland, Romania, Switzerland, Nigeria, Norway countries had relatively late research in this field. Additionally, we used Tableau Public software to illustrate the geographical distribution of publications across countries (Figure 5F). Among the top 10 countries, the data show a wide geographical representation: China and Japan are situated in Asia; the USA and Canada represent the Americas; Australia symbolizes Oceania; South Korea leads Africa in publication numbers; and Europe is represented by the UK, Netherlands, France, and Germany.

### 3.5 Analysis of journals and co-cited journals

**Figure 6:**
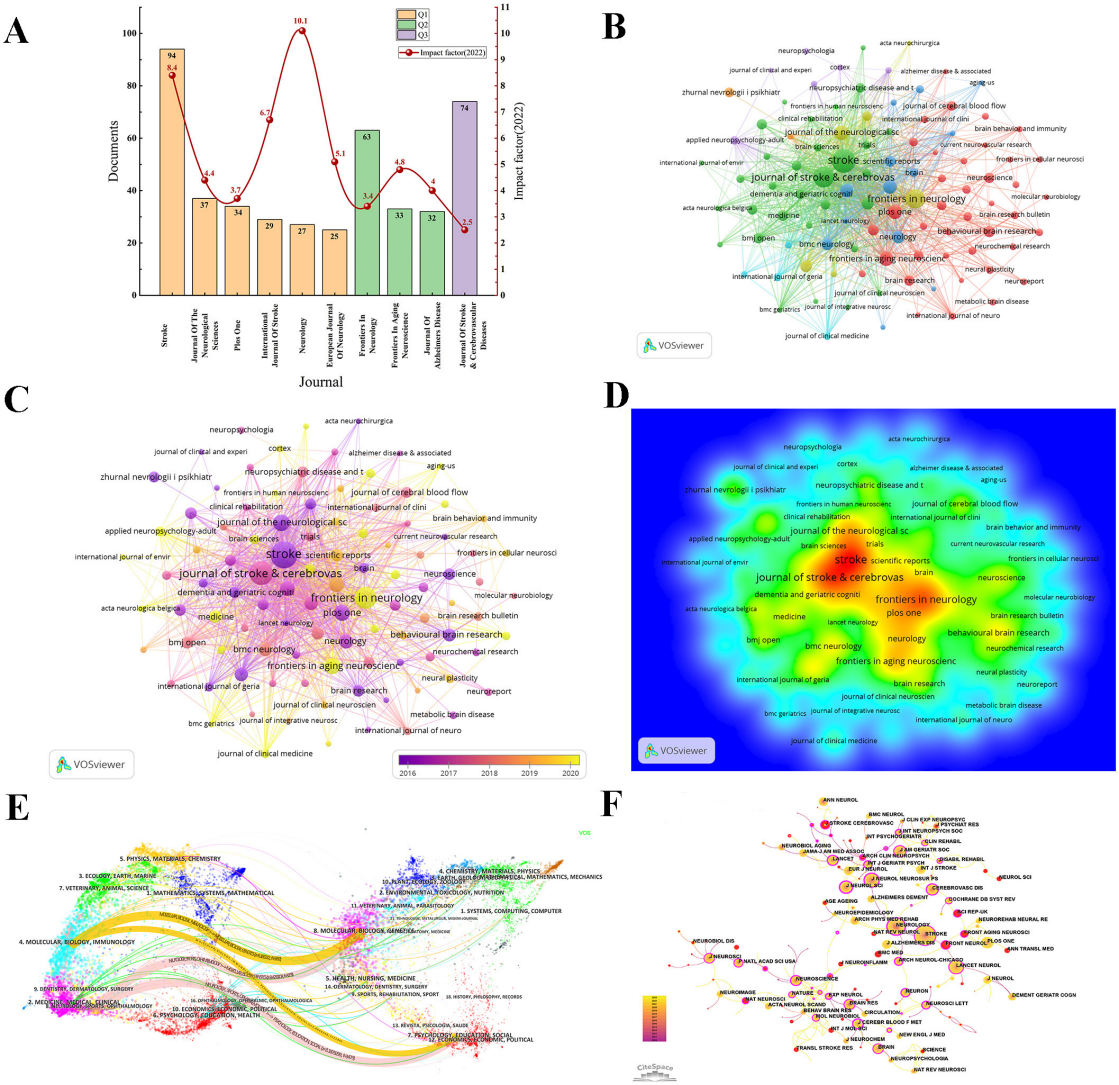
Analysis of journals and co-cited journals of PSCI publications. **(A)** Top 10 journals published PSCI publications and their IF. Bar graphs with the same color represent journals with the same JCR partition. **(B)** Network of journals based on VOSviewer. **(C)** Overlay visualization map of journals analysis. **(D)** The density map of journals. Red suggests high density, and the number of PSCI studies published in journals is positively connected to the level of density, the size of the words. **(E)** The journal dual-map overlay showcases the interconnections among various journals in the field of PSCI. **(F)** A visual map for CiteSpace network among co-cited journals. Co-cited journals refer to pairs of journals cited together by a third journal, forming a co-citation relationship. A high frequency of co-citation indicates that a journal is an essential theoretical foundation for the field's development.

Over the study period, 465 journals published articles related to the PSCI field, citing 7,233 journals. Among the top 10 journals, six were classified as Q1, 3 as Q2, and 1 as Q3 (Figure 6A), indicating the significant influence of PSCI research within the medical field. As shown in Supplementary Tables S2, S3, and Figure 6A, *Stroke* led in both publication volume (*n* = 94) and citations (*n* = 4,391), with a high impact factor (IF), establishing its authoritative influence in PSCI research (Q1, IF 2022 = 8.4). Other leading journals included the *Journal of Stroke & Cerebrovascular Diseases* (*n* = 74), *Frontiers in Neurology* (*n* = 64), *Journal of The Neurological Sciences* (*n* = 37), and *PLOS One* (*n* = 34). In comparison, the *Archives of Physical Medicine and Rehabilitation* had a lower publication output but the highest average number of citations (60.6), indicating significant potential. Conversely, the *Journal of Stroke & Cerebrovascular Diseases* had high publication and citation volumes but lower average citations per publication and a lower IF, suggesting that the journal has room for improvement in research quality.

Figures 6B–D illustrate the journal collaboration network, average publication time, and density of journal papers in PSCI research, respectively. Using VOSviewer, we identified 46 journals with 10 or more publications, all of which were part of interconnected networks. Among the top five prolific journals, *Frontiers in Neurology* stood out with a yellow hue, indicating the most recent average publication time and reflecting its status as a preferred journal for many researchers in recent years.

Using the dual-map overlay function of CiteSpace, Figure 6E displays the thematic distribution of journals. On the left are the citing journals in this field, while on the right are the cited journals. Different labels represent the disciplines covered by these journals. The *Z*-score is related to citation frequency, and the curved paths represent the citation relationships, indicating that publications in the journals on the left may cite publications from the journals on the right. We identified four main pathways in the figure, ranked in descending order by *Z*-scores (Supplementary Table S4). This analysis reveals that the most frequently covered subjects by citing journals in PSCI are molecular biology, immunology, neurology, sports, and ophthalmology. In contrast, the most cited subjects include molecular biology, genetics, psychology, education, and social sciences.

Figure 6F presents a co-cited journal visualization map, illustrating journals with significant foundational contributions to PSCI research. After adjusting the g-index (*k* = 15) and trimming the network, we obtained 482 nodes and 1,928 links. As shown in Table 3 and Figure 6F, *Stroke* is the most co-cited journal (co-citation = 1,690), followed by *Neurology* (co-citation = 1,111), *Lancet Neurology* (co-citation = 889), *Journal of Neurology Neurosurgery and Psychiatry* (co-citation = 780), and *PLoS One* (co-citation = 640). Notably, there was overlap between the top 10 most productive journals and the top 10 most co-cited journals, including *Stroke, Neurology, PLoS One*, and *Journal of The Neurological Sciences*. In terms of co-cited journal centrality (nodes represented by purple circles), *Neuroscience Letters* had the highest centrality (0.7), followed by *Neurochemistry International* (0.62), and *Neurology* (0.47).

**Table 3:** The top 10 co-cited journals in the field of PSCI in terms of citation frequency and centrality.

**Rank**	**Co-cited journal**	**Count**	**Centrality**	**JCR**	**IF (2022)**	**Co-cited Journal**	**Count**	**Centrality**	**JCR**	**IF (2022)**
1	Stroke	1,690	0.06	Q1	8.4	Neuroscience Letters	315	0.7	Q3	2.5
2	Neurology	1,111	0.47	Q1	10.1	Neurochemistry International	153	0.62	Q1	4.2
3	Lancet Neurology	889	0.05	Q1	48	Neurology	1,111	0.47	Q1	10.1
4	Journal of Neurology Neurosurgery and Psychiatry	780	0.47	Q1	11.1	Journal Of Neurology Neurosurgery and Psychiatry	780	0.47	Q1	11.1
5	PLos ONE	640	0	Q1	3.7	Archives of Neurology	362	0.43	–	–
6	Journal of The Neurological Sciences	629	0.1	Q1	4.4	Cerebrovascular Diseases	551	0.26	Q3	2.9
7	Journal of The American Geriatrics Society	603	0.2	Q1	6.3	Disability and Rehabilitation	201	0.26	Q1	2.2
8	Cerebrovascular Diseases	551	0.26	Q3	2.9	Clinical and Experimental Pharmacology and Physiology	12	0.26	Q2	2.9
9	Brain	515	0.16	Q1	15.3	Behavioural Brain Research	281	0.25	Q2	2.7
10	Lancet	505	0.13	Q1	168.9	Human Brain Mapping	135	0.24	Q1	4.8

### 3.6 Co-cited reference analysis

**Figure 7:**
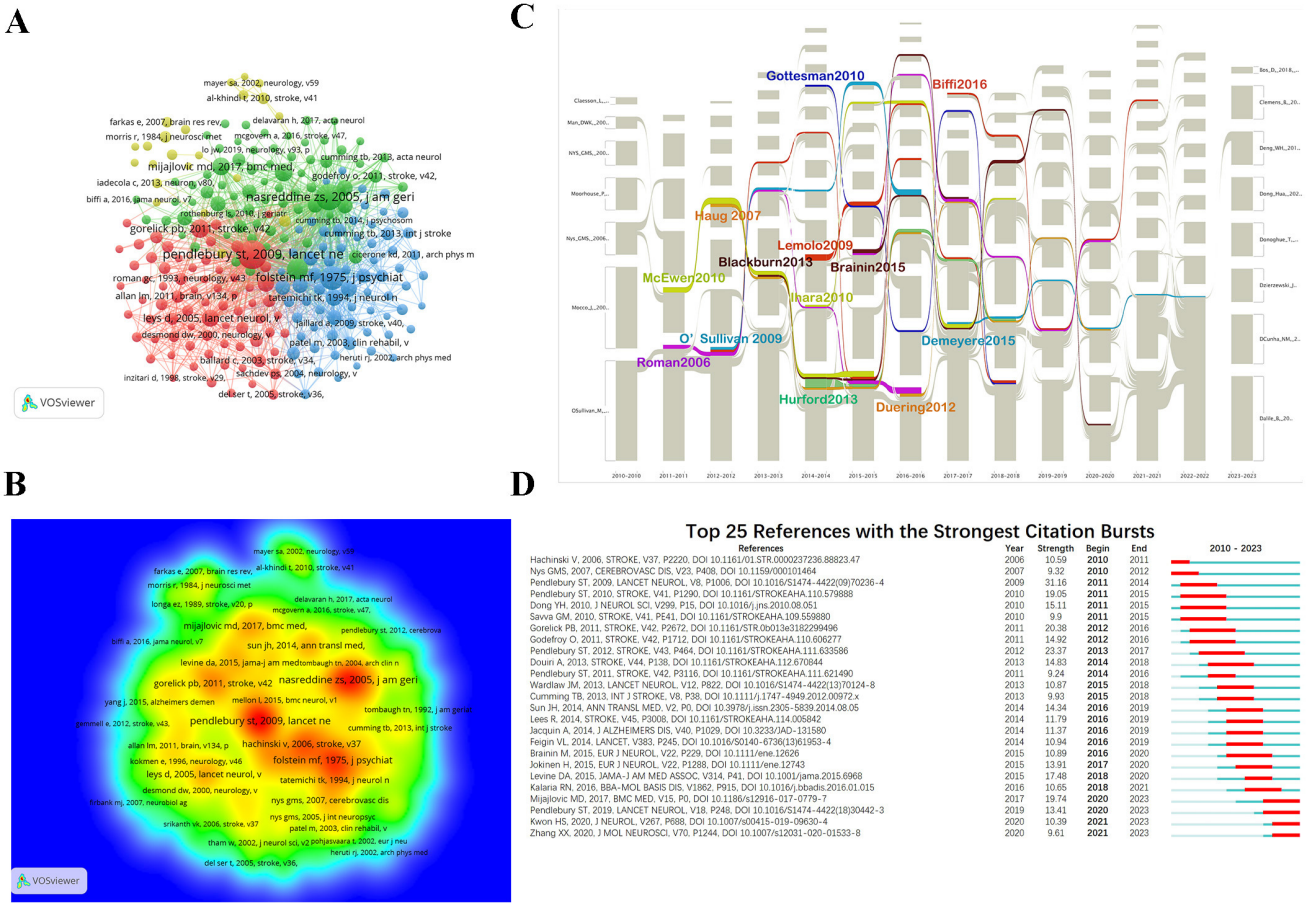
Visualization of co-cited references in PSCI research. **(A)** Reference co-citation network. Circles are co-cited literature. **(B)** Co-cited reference density visualization. **(C)** The alluvial flow graph of co-cited references. Each specific time point corresponds to a continuously evolving network structure. Each network consists of a number of clusters. The corresponding clusters in the adjacent network form a series of alluvial flows of how the same cluster evolves over time. **(D)** Top 25 references with the strongest citation bursts (sorted by the beginning year of burst). The red time period represents the duration of the outbreak, which can reflect the wide attention of scholars in the PSCI field during a certain period of time. The blue time period is divided into light blue and dark blue, in which light blue indicates the time when the literature does not appear during the study period, and dark blue refers to the cited time when the literature appears except for the outbreak time.

When two references are cited together by a third publication, they form a co-citation relationship, which plays a crucial role in the success of the third publication. The higher the citation frequency, the more the reference's research content is considered a foundational knowledge source in the field. The higher the centrality, the more the reference is seen as an inspiration, leading to more derivative research. Among the 53,566 co-cited references, 234 were cited at least 20 times (Figures 7A, B). During the study period, the top 10 most co-cited references were cited at least 105 times, accounting for ~3.36% of all co-cited references, indicating their irreplaceable influence (Table 4). Among them, Pendlebury et al.'s article (Pendlebury and Rothwell, 2009), published in *Lancet Neurology* (Q1; IF = 48), led the way in terms of citations (co-citation = 359). Other highly influential works included Nasreddine et al. (2005) (co-citation = 300), Folstein et al. (1975) (co-citation = 237), Hachinski et al. (2006) (co-citation =176), and Gorelick et al. (2011) (co-citation = 141) all published in Q1 journals and are considered crucial knowledge sources for future research in the field. Among the top 10 co-cited references, except for Sun et al.'s article, which was not indexed by the SCI, the remainder were published in Q1 journals. Among these journals, two were published in *Lancet Neurology*, three in *Stroke* (Q1, IF = 8.4), and the remaining four in the *Journal of The American Geriatrics Society* (Q1, IF = 6.3), *Journal of Psychiatric Research* (Q1, IF = 4.8), *BMC Medicine* (Q1, IF = 9.3), and *Journal of Neurology Neurosurgery and Psychiatry* (Q1, IF = 11.1).

**Table 4:** Top 10 co-cited references in the PSCI field.

**Rank**	**Author**	**Year**	**Co-cited references**	**DOI**	**Citations**	**Journal**	**JCR**	**IF (2022)**
1	Pendlebury, ST	2009	Prevalence, incidence, and factors associated with pre.stroke and post-stroke dementia: a systematic reviewand meta-analysis	10.1016/s1474-4422(09)70236-4	359	Lancet Neurology	Q1	48.0
2	Nasreddine ZS	2005	The Montreal Cognitive Assessment, MoCA: a brief screening tool for mild cognitive impairment	10.1111/j.1532-5415.2005.53221.x	300	Journal of The American Geriatrics Society	Q1	6.3
3	Folstein MF	1975	“Mini-mental state”. A practical method for grading the cognitive state of patients for the clinician	10.1016/0022-3956(75)90026-6	237	Journal of Psychiatric Research	Q1	4.8
4	Hachinski V	2006	National Institute of Neurological Disorders and Stroke-Canadian Stroke Network vascular cognitive impairment harmonization standards	10.1161/01.str.0000237236.88823.47	176	Stroke	Q1	8.4
5	Gorelick PB	2011	Vascular contributions to cognitive impairment and dementia: a statement for healthcare professionals from the american heart association/american stroke association	10.1161/str.0b013e3182299496	141	Stroke	Q1	8.4
6	Mijajlovié MD	2017	Post-stroke dementia—a comprehensive review	10.1186/s12916-017-0779-7	127	BMC Medicine	Q1	9.3
7	Sun JH	2014	Post-stroke cognitive impairment: epidemiology, mechanisms and management	10.3978/j.issn.2305-5839.2014.08.05	123	Ann Transl Med	–	–
8	Leys D	2005	Poststroke dementia	10.1016/s1474-4422(05)70221-0	120	Lancet Neurology	Q1	48
9	Adams HP Jr	1993	Classification of subtype of acute ischemic stroke. Definitions for use in a multicenter clinical trial. TOAST. Trial of Org 10172 in Acute Stroke Treatment	10.1161/01.str.24.1.35	113	Stroke	Q1	8.4
10	Tatemichi Tk	1994	Cognitive impairment after stroke: frequency, patterns, and relationship to functional abilities	10.1136/jnnp.57.2.202	105	Journal of Neurology Neurosurgery and Psychiatry	Q1	11.1

Using CiteSpace 5.7.R5, we generated a series of co-cited reference networks, which were then input into the alluvial generator. After filtering and adjusting the colors, we obtained an alluvial flow graph of the cited references (Figure 7C). The co-cited references with the longest-lasting streams are listed in Table 5, with the works by Brainin et al. (2015b), Demeyere et al. (2015), and Román and Kalaria (2006) demonstrating the longest flow durations, lasting up to 6 years. The longer the stream lasted, the longer the reference was cited, indicating sustained recognition by PSCI researchers. Additionally, 10 studies had streams lasting 5 years, with Hurford et al.'s (2013) reference having the widest stream, suggesting a significant impact on PSCI research frontiers.

**Table 5:** Co-cited references with long duration in the alluvial flow graph.

**Rank**	**Author**	**Year**	**Title**	**Time of duration**	**Journal**	**JCR**	**IF (2022)**
1	Brainin M	2015	Prevention of poststroke cognitive decline: ASPIS–a multicenter, randomized, observer-blind, parallel group clinical trial to evaluate multiple lifestyle interventions–study design and baseline characteristics	6	International Journal of Stroke	Q1	6.7
2	Roman GC	2006	Vascular determinants of cholinergic deficits in Alzheimer disease and vascular dementia	6	Neurobiology of Aging	Q2	4.2
3	Demeyere N	2015	The Oxford Cognitive Screen (OCS): validation of a stroke-specific short cognitive screening tool	6	Psychological Assessment	Q1	3.6
4	Gottesman RF	2010	Predictors and assessment of cognitive dysfunction resulting from ischaemic stroke	5	Lancet Neurology	Q1	48
5	Biffi A	2016	Risk Factors Associated with Early vs Delayed Dementia After Intracerebral Hemorrhage	5	Jama Neurology	Q1	29
6	Ihara M	2010	Quantification of myelin loss in frontal lobe white matter in vascular dementia, Alzheimer's disease, and dementia with Lewy bodies	5	Acta Neuropathologica	Q1	12.7
7	Duering M	2012	Incident subcortical infarcts induce focal thinning in connected cortical regions	5	Neurology	Q1	10.1
8	Lemolo F	2009	Pathophysiology of vascular dementia	5	Immunity & Ageing	Q1	7.9
9	Blackburn DJ	2013	Cognitive screening in the acute stroke setting	5	Age and Ageing	Q1	6.7
10	Hurford R	2013	Domain-specific trends in cognitive impairment after acute ischaemic stroke	5	Journal of Neurology	Q1	6
11	Haug T	2007	Cognitive outcome after aneurysmal subarachnoid hemorrhage: time course of recovery and relationship to clinical, radiological, and management parameters	5	Neurosurgery	Q1	4.8
12	O'Sullivan M	2009	Hippocampal volume is an independent predictor of cognitive performance in CADASIL	5	Neurobiology of Aging	Q2	4.2
13	McEwen SE	2010	There's a real plan here, and I am responsible for that plan': participant experiences with a novel cognitive-based treatment approach for adults living with chronic stroke	5	Disability and Rehabilitation	Q1	2.2

Figure 7D presents the 25 most co-cited references that received significant attention between 2012 and early 2023. By examining the corresponding burst strengths over different periods, we can identify trends in the research focus, helping discover emerging trends in recent years. The figure shows that the studies by Mijajlović et al., Pendlebury et al., Kwon et al., and Zhang et al. have received significant attention in recent years, primarily focusing on risk factors for PSCI (Pendlebury and Rothwell, 2019), objective diagnostic methods (Zhang and Bi, 2020), prognosis (Kwon et al., 2020), and summary reviews (Mijajlović et al., 2017), providing essential foundations for cutting-edge hotspots. Moreover, we found that Pendlebury et al.'s study (Pendlebury and Rothwell, 2009) was of great significance in the field of PSCI (strength = 31.16). Their findings indicated that stroke itself significantly and directly affects the occurrence of dementia, surpassing the influence of vascular risk factors. Furthermore, an increasing number of scholars are exploring new trends through comprehensive reviews.

### 3.7 Keyword analysis

**Figure 8:**
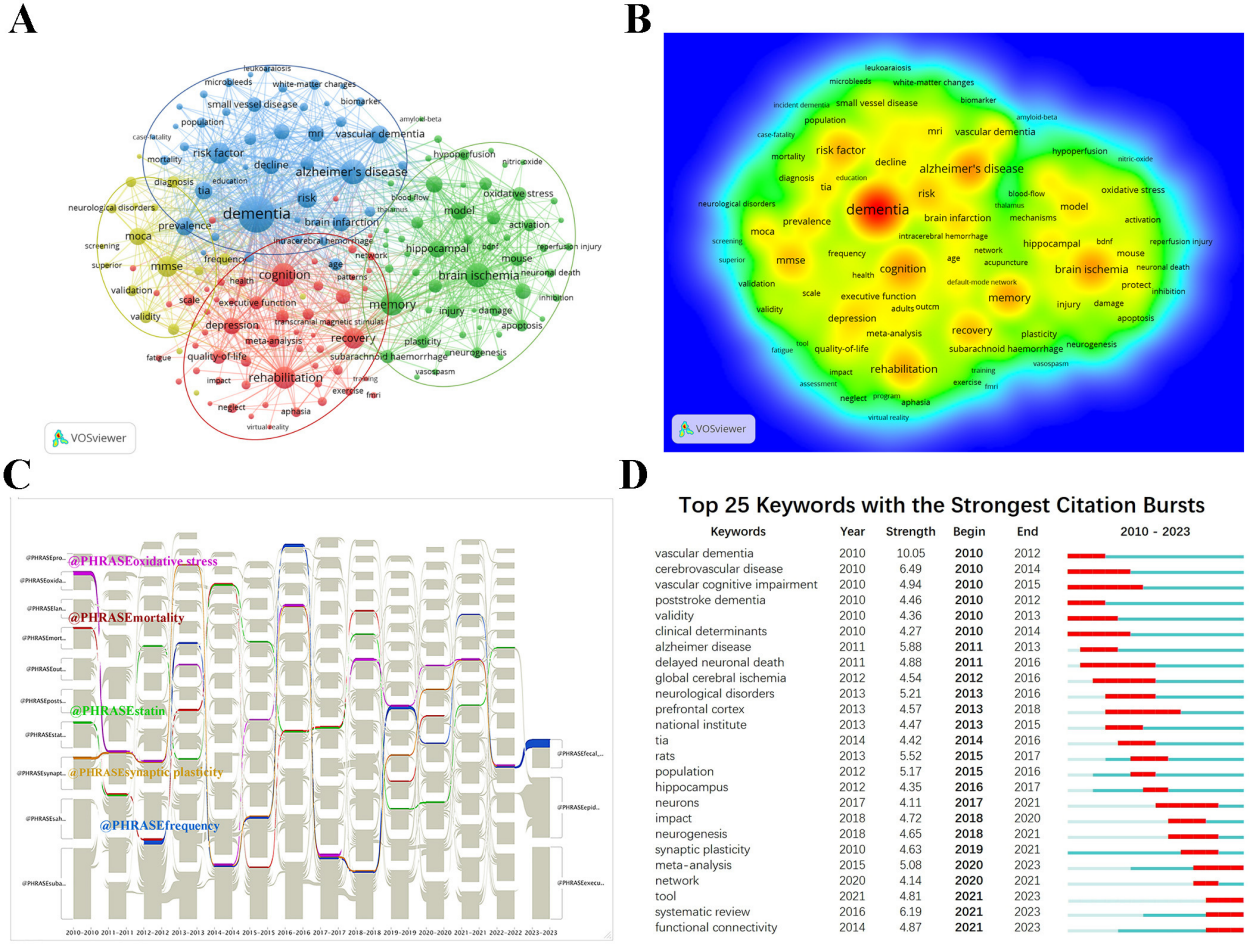
Bibliometric analysis of keywords in the PSCI field. **(A)** The keyword co-occurrence map. Nodes with the same color belong to the same cluster. The 178 keywords fell into four clusters based on colors: Cluster 1, 2, 3, and 4 are, respectively, red, green, blue and yellow. The node size denotes the occurrence frequency. And more lines between nodes represent stronger associations between terms **(B)** The keyword density map. **(C)** The alluvial flow graph of keywords. **(D)** Top 25 keywords with the strongest citation bursts.

We used VOSviewer to analyze the keywords extracted from 1,934 publications, which included 5,698 terms. As shown in Figures 8A, B, and Supplementary Table S5, the keyword “dementia” is the most frequently occurring term (588 occurrences), followed by “Alzheimer's disease” (271 occurrences), “brain ischemia” (248 occurrences), “cognition” (246 occurrences), “memory” (229 occurrences), “rehabilitation” (208 occurrences), and “risk factor” (201 occurrences), among others.

During the analysis, 178 keywords appeared at least 12 times and were grouped into four main categories (Figure 8A). Each color represents a cluster, with larger node circles indicating higher keyword frequency. As shown in Figure 8A, we conduct a specific analysis based on the clustering from the largest to the smallest. Cluster one (red) focuses on thecc of PSCI and includes the most keywords, with top terms such as “cognition,” “rehabilitation,” “recovery,” “depression,” “quality of life,” “executive function,” “anxiety,” “aphasia,” “neglect,” “treatment,” and “exercise.” Cluster two (green) mainly involves pathogenesis and covers terms such as “brain ischemia,” “memory,” “model,” “hippocampal,” “inflammation,” “protect,” “oxidative stress,” “injury,” and “plasticity.” The top 10 keywords related to the mechanism are shown in Supplementary Table S6. Cluster three (blue) emphasizes PSCI risk factors, including keywords such as “dementia,” “Alzheimer's disease,” “risk factor,” “vascular dementia,” “TIA (transient ischemic attack),” “brain infarction,” “MRI,” “small vessel disease,” “vascular cognitive impairment,” and “age.” The smallest cluster (yellow) primarily relates to assessment and diagnosis, covering terms such as “MMSE,” “MoCA (Montreal Cognitive Assessment),” “validity,” “diagnosis,” “frequency,” “reliability,” “validation,” “neurological disorders,” “neuropsychology,” and “questionnaire.”

To explore the research topics that have captured attention from 2010 to early 2023, we used CiteSpace in conjunction with the alluvial generator to successfully plot the alluvial flow graph of PSCI's academic keywords (Figure 8C). The keywords with the longest-lasting streams are listed in Supplementary Table S7, with “synaptic plasticity,” “statin,” and “oxidative stress” having the longest streams from 2010 to 2022. This suggests that pathogenesis, treatment, and prevention in PSCI have been critical research topics over the past 13 years. Additionally, the assessment and diagnosis of PSCI have also been widely researched.

To better understand the research trends and hot topics in the PSCI field, we utilized CiteSpace's keyword burst analysis function. The results, as shown in Figure 8D, indicate that the emergence of keywords reflects a rapid increase in frequency over a short period, marking them as hot topics of academic interest. The year 2010 saw the highest citation bursts for keywords such as “vascular dementia,” “cerebrovascular disease,” “vascular cognitive impairment,” “poststroke dementia,” and “validity,” indicating an early focus on PSCI's vascular risk factors and assessment. Research then shifted toward mechanisms, with bursts including “delayed neuronal death,” “global cerebral ischemia,” “rats,” “neurological disorder,” “hippocampus,” “neurons,” and “neurogenesis.” In the past 5 years, keywords such as “synaptic plasticity,” “meta-analysis,” “network,” “tool,” “systematic review,” and “functional connectivity” have frequently appeared.

### 3.8 Analysis of CSVD-related research in PSCI

**Figure 9:**
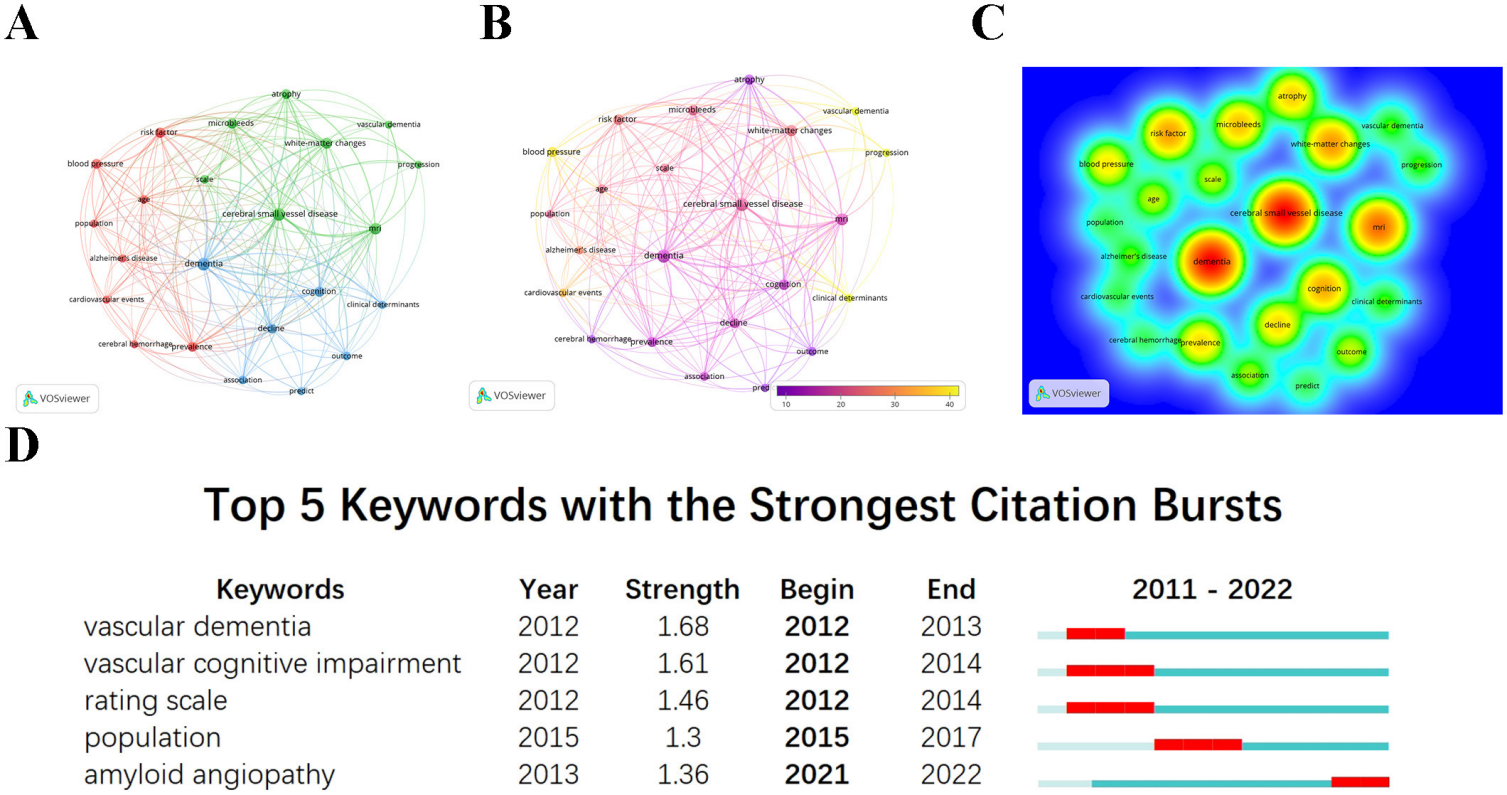
Visualization analysis of keywords in CSVD research related to PSCI. **(A)** Co-occurrence network of keywords based on VOSviewer. **(B)** Overlay visualization map of keywords analysis. Keywords in yellow occurred later than those in purple. **(C)** The density map of keywords. The level of density, the size of nodes and words all reflect the co-occurrence frequencies. **(D)** Top five keywords with the strongest citation bursts.

As shown in Figure 8D, many keywords that appeared around 2010 were related to cerebrovascular diseases. Cerebral infarction caused by CSVD accounts for 25–50% of all strokes, which is relatively higher than that in Western countries (Tsai et al., 2013; Georgakis et al., 2023). CSVD is a crucial pathological basis for cognitive impairment and dementia, significantly increasing the risk of PSD (Lam et al., 2021). We screened the retrieved literature and reanalyzed 56 papers related to CSVD to further explore the focus of CSVD-related studies in PSCI. Using VOSviewer, we analyzed all keywords and identified 23 keywords that appeared at least four times, as shown in Figures 9A–C. As shown in Figures and Table 6, aside from CSVD, “dementia” appears with the highest frequency, followed by “MRI.” MRI is well-known as the most crucial neuroimaging examination method in PSCI (Mijajlović et al., 2017), with typical imaging features including lacunar infarcts, WMHs, CMBs, perivascular spaces, and cerebral atrophy (Du and Xu, 2019; Inoue et al., 2023; Markus and Erik de Leeuw, 2023). Furthermore, imaging marker burdens and severity can reflect cognitive and functional outcomes after stroke (Georgakis et al., 2023). Notably, hypertension is the most important risk factor for non-amyloid CSVD, and primary prevention data have demonstrated that strict control of hypertension may delay cognitive dysfunction (Markus and de Leeuw, 2023). Looking ahead, keyword burst analysis (Figure 9D) suggests that CAA could represent a new research trend in the future.

**Table 6:** The top 20 keywords with the highest frequency in CSVD studies related to PSCI.

**Rank**	**Keywords**	**Occurrences**	**Average citations**	**Rank**	**Keywords**	**Occurrences**	**Average citations**
1	Cerebral small vessel disease	38	20.79	11	Blood pressure	10	37.7
2	Dementia	38	15.34	12	Age	7	23.29
3	MRI	22	17.64	13	Scale	7	23
4	White-matter changes	17	23.76	14	Outcome	7	1.86
5	Risk factor	16	24.5	15	Association	6	15.17
6	Cognition	15	14.6	16	Vascular dementia	5	45.8
7	Microbleeds	13	22.54	17	Clinical determinants	5	43
8	Atrophy	12	11.67	18	Progression	5	40.2
9	Decline	11	16.73	19	Alzheimer's disease	5	27.4
10	Prevalence	11	14.73	20	Cardiovascular events	4	32.25

## 4 Discussion

### 4.1 Basic information

This study conducted a comprehensive bibliometric analysis of the PSCI field from 2010 to early 2023. The research suggests that the annual number of publications is still on the rise as a whole, indicating that the PSCI field has a broad research prospect in the future. Among the co-cited authors, Pendlebury has been cited the most frequently and is a leading figure in the field of PSCI. China not only has the largest number of publications, but also has the largest number of top 10 institutions, which is mainly related to the increasing importance of PSCI in China in recent years. China has close cooperation with the United States, and the United Kingdom has the largest number of cooperative countries. Nevertheless, there is still a need to strengthen international cooperation in order to achieve wider academic exchanges. Stroke is the journal with the largest number of publications and co-citations, and has a high academic level and international influence.

### 4.2 Focus and frontiers

Two previous bibliometric studies on PSCI visualized relevant data using bibliometric software (Chi et al., 2023; Ou et al., 2023); however, they did not fully discuss a keyword cluster analysis in a comprehensive and systematic manner. In contrast, keyword analysis was at the core of this study. Additionally, we combined the current research hotspots and performed a keyword analysis of CSVD-related articles within the PSCI to thoroughly investigate the significant outcomes between them.

#### 4.2.1 Risk factors for PSCI

Several risk factors are associated with PSCI (Desmond et al., 2000; Walters et al., 2003; Pendlebury and Rothwell, 2009, 2019; Wang et al., 2021; Rost et al., 2022; El Husseini et al., 2023). The key susceptibility factors include degenerative diseases, cerebrovascular diseases, and age, among others (El Husseini et al., 2023). Desmond et al. (2000) found that 16.3% of patients with PSD had dementia before onset. Furthermore, some studies have revealed that there is a significant overlap between PSCI, VCI, and AD in terms of neuropathology and biochemical mechanisms (Gemmell et al., 2012, 2014; Mok et al., 2017). Firbank et al. (2007) found that some characteristic features of AD, such as temporal lobe atrophy and amyloid deposition, are associated with cognitive decline in PSD patients (Mok et al., 2017). These findings highlight the substantial impact of neurodegenerative diseases on the cognitive function of stroke patients.

A history of cerebral infarction is a key predictor of PSCI (Shim, 2014), and stroke-related features are primarily associated with early-onset dementia in stroke patients (Mok et al., 2017). For example, Pendlebury et al. reported that the incidence of dementia after recurrent stroke is approximately three times that after a first-time stroke, with each recurrence leading to a cumulative increase in the incidence of dementia (Pendlebury and Rothwell, 2009). Additionally, the size and location of the infarct foci are significantly associated with PSD (Pendlebury and Rothwell, 2009; Rost et al., 2022). Moreover, CSVD is a major cause of stroke, and one study suggested that the CSVD burden is an important predictor of hemorrhagic PSD (El Husseini et al., 2023). Late-onset stroke dementia, in particular, is primarily associated with CSVD rather than recurrent stroke or AD pathology, and the correlation strengthens as the CSVD burden increases (Mok et al., 2017). Finally, advanced age is also an important risk factor for cognitive decline in stroke patients (Pendlebury and Rothwell, 2009; Brainin et al., 2015a; Wadley et al., 2018; Wang et al., 2021; Rost et al., 2022). A study demonstrated that each 1-year increase in baseline age was associated with 17% higher odds of cognitive impairment per year during follow-up (Rost et al., 2022).

#### 4.2.2 Pathogenesis of PSCI

The specific mechanisms underlying PSCI remain unclear and involve several factors, including cerebrovascular injury, neurodegenerative processes, genetic influences, inflammation, and molecular changes (Li et al., 2022). The primary mechanism of PSCI is the disruption of brain neuroanatomical structures due to cerebrovascular lesions. These lesions can cause brain ischemia, leading to neuronal death or nerve fiber damage, which hinders information transmission within neural networks and ultimately impairs cognitive function (Zuo et al., 2017). This impairment is associated with the location of ischemia. Some studies have indicated that brain ischemia and hypoperfusion can result in a selective reduction in hippocampal volume (Gemmell et al., 2012, 2014), contributing to hippocampal atrophy and cognitive impairments, particularly in memory. Furthermore, when vascular injury coincides with neurodegenerative diseases, hippocampal atrophy is more pronounced, leading to more substantial cognitive decline (Gemmell et al., 2014). In terms of genetics, the ApoEε4 allele is widely recognized as a risk factor for Alzheimer's disease, and some studies suggest a possible connection to PSCI. This association may involve cognitive decline related to its effects on lipid metabolism, atherosclerosis, synaptic plasticity, and neuronal cell function recovery (Zuo et al., 2017).

The molecular mechanisms mainly include damage to the cholinergic transmission pathway, excitotoxicity, and oxidative stress (Zuo et al., 2017; Maida et al., 2020; Li et al., 2022). Neuronal ischemia leads to excessive glutamate release, resulting in excitotoxic cell death. Glutamate receptor activation can also indirectly activate free radicals (Mishra and Hedna, 2013). Moreover, neuronal damage produces reactive oxygen species, depleting antioxidants such as glutathione and reducing the clearance of oxidative free radicals. This depletion contributes to cell death due to oxidative stress (Zuo et al., 2017; Maida et al., 2020). The next step involves the activation of microglia and astrocytes, which release chemokines, cytokines, and NO, inducing leukocyte migration to the ischemic area and attracting other immune cells to the region. This response results in increased neuronal death and ultimately enlarges the infarct size (Mishra and Hedna, 2013). Many studies have demonstrated that elevated concentrations of inflammatory markers, such as C-reactive protein, interleukin (IL), tumor necrosis factor (TNF), interferon (IFN), and complement, lead to poorer cognitive outcomes (Rothenburg et al., 2010; Shim, 2014; Narasimhalu et al., 2015; Sandvig et al., 2023). Overall, increasing research has suggested that reducing inflammation serves as a neuroprotective mechanism in stroke, aiming to improve cerebrovascular injury. Nevertheless, while these findings show promising applications in animal models, further exploration in clinical practice is required (Mishra and Hedna, 2013).

Currently, “functional connectivity” are research hotspots, attracting widespread attention from scholars. In particular, enhancing synaptic plasticity and interhemispheric functional connectivity in the brain can reduce stroke-related damage and improve cognitive function (Naseh et al., 2022; Wang et al., 2022; Yang et al., 2022). Moreover, transcranial direct current stimulation (tDCS) is a promising tool for cognitive rehabilitation, known to improve cognitive impairment by increasing interhemispheric functional connectivity (Yang et al., 2022). Therefore, alterations in functional connectivity play important roles in the pathogenesis and treatment of PSCI, which still require further investigation through animal experiments.

#### 4.2.3 Assessment and diagnosis of PSCI

Cognitive assessment in the acute phase of stroke can predict the occurrence of PSCI. Current guidelines in China recommend cognitive screening for acute-phase patients unless they are unconscious or unresponsive (Wang et al., 2021). Moreover, PSCI is a dynamic process characterized by considerable heterogeneity, with cognitive impairment often becoming prominent 3 months post-stroke, making early and regular assessments of cognitive function over time essential (Wang et al., 2021). Assessments primarily involve neuropsychological evaluations, activities of daily living assessments, and evaluations of psychiatric and behavioral symptoms (Wang et al., 2021). The MMSE and MoCA are currently the most widely studied cognitive screening tools, with the MoCA considered superior to the MMSE (El Husseini et al., 2023). Considering that compared with AD, PSCI has a more significant impact on other cognitive domains other than memory. Therefore, when cognitive impairment is detected during screening, we need to conduct a series of standardized neuropsychological assessments to further help identify cognitive domains that are impaired beyond memory (El Husseini et al., 2023). Additionally, differences in neuropsychological testing tools are inevitable across countries, studies, and clinical settings. Thus, the effectiveness and usability of different screening methods require further validation in various languages while also considering time and cost constraints (Shim, 2014).

In the keyword burst analysis, the term “tool” has shown a sudden rise in frequency in recent years. As research on PSCI continues to develop, there is growing international interest in utilizing screening tools to identify cognitive impairment early, before it affects quality of life. Additionally, predictive models are being created to assess the risk of cognitive impairment worsening in the future (Hbid et al., 2021; Cova et al., 2022), which can facilitate better rehabilitation planning and anticipate long-term post-stroke outcomes. In the future, we can also develop in this direction.

#### 4.2.4 Treatment and rehabilitation of PSCI

According to the keyword co-occurrence analysis, the red cluster primarily highlights the treatment and rehabilitation of PSCI. This is particularly relevant, as stroke survivors with cognitive impairment face higher mortality rates, a poorer quality of life, and familial and social health issues (Wang et al., 2021). This makes the early detection and management of PSCI a critical concern. The consensus indicates that PSCI treatment mainly addresses cognitive function, psychiatric and behavioral symptoms, and activities of daily living (Wang et al., 2021).

As far as cognitive function is concerned, cholinesterase inhibitors have been shown to improve cognitive performance (Birks and Craig, 2006; Barrett et al., 2011; Kim et al., 2020). In terms of post-stroke aphasia, Berthier et al. (2009) pointed out that memantine can reduce the severity of aphasia, particularly when combined with constraint-induced aphasia therapy. Additionally, tDCS has been found to be effective in treating post-stroke aphasia (El Husseini et al., 2023). Furthermore, cognitive strategy training has been shown to be effective in recovering executive function after a stroke, although the exact extent of recovery and the specific mechanisms involved remain to be investigated (Cramer et al., 2023).

Beyond cognitive impairments, psychiatric symptoms are also a major concern for patients with PSCI, with depression being particularly common. Notably, severe psychiatric symptoms can pose safety threats to both the patient and others (Wang et al., 2021), making the treatment of these symptoms crucial. In this context, studies have shown that improving psychiatric symptoms, such as depression and anxiety, can enhance cognitive function (Shim, 2014; Mijajlović et al., 2017), regardless of the drug's mechanism (Haring, 2002). In summary, various prevention, treatment, and rehabilitation methods exist for PSCI, and active intervention can reduce the burden on families as well as alleviate social pressure.

#### 4.2.5 Analysis of CSVD-related research in PSCI

The most common pathological basis of vascular cognitive impairment and dementia is CSVD (Inoue et al., 2023). On MRI, CSVD manifests as WMHs, lacunes, perivascular spaces, CMBs, brain atrophy, and other chronic pathological changes that weaken neurons and diminish synapses' resistance to brain injury, reducing the brain's resilience to vascular damage (Mok et al., 2017). Based on the results of this study's analysis, we will focus on discussing the impact of WMHs and CMBs on cognition.

Both CMBs and WMHs are vascular injury markers that promote cognitive impairment in ischemic stroke patients (Tang et al., 2011). Notably, the total volume and lesion location of WMHs are independently associated with PSCI. For instance, subcortical lacunar infarcts double the risk of PSCI, and when accompanied by severe WMHs, the risk increases 11-fold. Similarly, CMBs are a significant predictor of hemorrhagic PSD (El Husseini et al., 2023). In a multivariate analysis, Gregoire et al. (2013) found that after adjusting for hypertension and WMHs, lobar microbleeds were still associated with impaired executive function in ischemic stroke patients. A long-term longitudinal study revealed that, compared to PSCIND patients with CMBs, the absence of CMBs can predict reversible cognitive recovery, with different CMB locations affecting recoveries in distinct cognitive domains, although the specific mechanisms remain unclear (Tang et al., 2011). Finally, there is a link between WMHs and CMBs. Regardless of the specific location of CMBs, WMHs volume was associated with CMBs, with the strongest correlation observed for deep/infratentorial CMBs (Poels et al., 2010). Importantly, the severity of white matter changes increases with the CAA-related CMB burden (Gregoire et al., 2011).

Based on the results of this study, hypertension is a significant risk factor for PSCI. Hypertension is also the most important risk factor for non-amyloid SVD (Markus and de Leeuw, 2023). Moreover, high blood pressure variability is also a risk factor for PSCI. As shown by Kim et al. (2021), the greater the blood pressure variability, the faster the cognitive decline during follow-up in ischemic stroke patients. The underlying mechanism likely involves hypertension-induced cerebral microvascular dysfunction, leading to WMHs, lacunes, and CMBs, which further impair cognitive function (Uiterwijk et al., 2017). In addition, hypertension may disrupt connections in the temporal lobe, thalamus, and prefrontal cortex, especially in the hippocampus, leading to cognitive decline (Lee et al., 2021).

Many studies have shown that hypertension is associated with WMHs (Haring, 2002). Interestingly, the relationship between hypertension and WMHs may follow a “J” curve, where both low and high blood pressure, increased blood pressure fluctuations, and smaller declines in nocturnal blood pressure are associated with increased WMHs (Mok and Kim, 2015). The main mechanism involves hypertension's impact on deep perforating arteries, leading to atherosclerosis, inducing lipid hyalinosis, and promoting vascular remodeling (Iadecola et al., 2009). This process makes blood vessels prone to hypoperfusion during low blood pressure (Manolio et al., 2003). Hypertension also exacerbates the twisting and bending of these perforating arteries (Low et al., 2019), increasing resistance to blood flow, which predisposes individuals to hypoperfusion and, consequently, to WMHs. Hypoperfusion may result in the selective collapse of key proteins in the paranodal axon-glial junctions, thereby affecting white matter function (Kim et al., 2021). Thus, hypertension can lead to WMHs by causing hypoperfusion, which in turn impairs cognitive function. In addition to these mechanisms, WMH formation is also related to blood–brain barrier disruption, plasma protein infiltration into the vessel wall and surrounding brain parenchyma (Mok and Kim, 2015), and periventricular venous collagen disease (Moody et al., 1995).

Additionally, hypertension is closely related to CMBs. The primary cause of deep microbleeds is damage to the deep perforating arteries, resulting from increased blood flow velocity due to hypertension (Jung et al., 2020). Prolonged hypertension leads to atherosclerosis and lipid hyalinosis in the vasculature of the perforating arteries (Iadecola et al., 2009), which affects the vascular wall structure of small perforating arterioles, reducing vascular elasticity and potentially promoting the formation of microaneurysms. These microaneurysms may ultimately rupture and lead to the development of CMBs (Rosenblum, 2008). The mechanism behind lobar microbleeds is primarily related to CAA (Gregoire et al., 2013). In this case, amyloid-β deposits in the small artery walls cause endothelial and smooth muscle dysfunction, resulting in vessel wall thickening. These vascular changes make the vessels fragile, leading to microaneurysm formation and leakage (Gregoire et al., 2011). Additionally, local blood flow regulation is impaired, increasing the likelihood of small vessel occlusion and resulting in local ischemia (Gregoire et al., 2011). This may explain why WMHs worsen as CMB lesions increase. When the CMBs is mixed-type (both in the cerebral lobe and deep areas), some studies have suggested that the mechanism is mainly attributed to hypertensive vasculopathy (Jung et al., 2020). Moreover, hypertensive vasculopathy and CAA do not occur independently in PSCI. Following hypertensive damage to small cerebral vessels, the clearance of β-amyloid is further reduced, leading to increased deposition of β-amyloid in the vessel walls, which exacerbates cognitive impairment (Ding et al., 2017). Furthermore, CMBs have also been found to increase the risk of PSD independently of the number and type of acute cerebral infarctions. This suggests that CMBs may impair cognitive function through direct damage to surrounding brain tissue (Yatawara et al., 2020). The observation of tissue necrosis in histopathological studies supports this notion (Gregoire et al., 2013). Therefore, the results of this study suggest that blood pressure control plays a crucial role in reducing the incidence of cognitive impairment.

Through this study, we found that CAA-related research still has great potential for development. Stroke can promote CAA and dementia by inducing amyloid β deposition (Goulay et al., 2020; Rost et al., 2022), and the specific neuropathological basis and pathophysiological mechanism remain to be studied. We can predict cognitive decline in patients with hemorrhagic and ischemic stroke by CAA-related specific MRI scores (Pasi et al., 2021; Sagnier et al., 2021), and the specific explanation may require a lot of evidence. In addition, the CSVD overall score may be superior to the CAA-related specific score in predicting PSCI, suggesting that most ICH survivors had some degree of mixed CSVD (Pasi et al., 2021). CAA may aggravate cognitive decline in stroke survivors through the following mechanisms, such as blood-brain barrier damage (Gatti et al., 2020), inflammatory response (Ono and Tsuji, 2020), direct toxic effect of amyloid β (Zott et al., 2019), vascular damage (Gregoire et al., 2011) and so on. But in the future, more studies are needed to further elucidate the neuropathological basis and pathophysiological mechanisms related to the development of PSCI (Rost et al., 2022). In the future, we can further study PSCI and CAA to guide the prevention of stroke and provide information for further research to improve the long-term prognosis of stroke.

Bibliometrics can assist clinical research in various ways. First, through bibliometric methods, we have highlighted key research areas in PSCI, such as risk factors, pathogenesis, evaluation and diagnosis, treatment and rehabilitation. Specifically, hypertension has been identified as a key factor in the formation of WMH and CMBs, further aggravating cognitive dysfunction. These findings suggest that doctors can reduce the incidence of PSCI by controlling blood pressure in treatment decisions, emphasizing early prevention, providing a scientific basis for clinical diagnosis and treatment, and promoting the rapid and comprehensive development of PSCI. Second, bibliometrics helps identify emerging research trends. Future studies should focus on areas such as functional connectivity, and the role of CAA in the mechanisms involved to identify effective ways to improve cognition and achieve better prevention and treatment outcomes, thereby reducing family and social burdens. Moreover, clinicians can explore tools to identify early cognitive impairment in stroke patients across different environments and develop PSCI risk prediction tools for more targeted strategies. Additionally, scholars can conduct comprehensive analyses of the research status of PSCI through various statistical forms to understand the latest trends and avoid redundant research. By integrating advancements in PSCI research into clinical practice, clinicians and researchers can refine research directions and strategies, ultimately enhancing the quality and impact of their findings. Finally, this study identifies the most influential countries, institutions, authors, and journals through visual analysis, which can guide junior researchers in identifying mentors and partners when submitting contributions, selecting appropriate journals and institutions, and seeking suitable national collaborations to make informed decisions on the allocation of funds and resources.

### 4.3 Limitations and strengths

First, to ensure data quality and completeness, the data in this study were obtained exclusively from the WoSCC database. Although the Web of Science database is widely recognized by bibliometric researchers, it cannot cover all relevant studies in this field, which poses a risk of overlooking research from other databases. Additionally, data selection in this study relied on manual filtering to exclude literature unrelated to PSCI, which may introduce bias or omissions. Furthermore, the search language was limited to English publications, inevitably overlooking studies in other languages. Thus, while our search strategy aimed to cover all possible expressions to ensure comprehensive inclusion, there may still be some omissions. Finally, some recently published high-quality articles may have lower citation counts due to their short publication time, necessitating further bibliometric studies in the future to update data and explore new trends and hotspots in PSCI research.

Despite the aforementioned limitations, this study has several advantages. For instance, it combines PSCI with the emerging research hotspot of CSVD for the first time. The visualization results provide valuable information for global PSCI researchers, helping them identify new research directions and hotspots, thereby promoting rapid and efficient development in this field. Additionally, based on our findings and previous clinical studies, we reaffirm the important role of hypertension in PSCI and further explore the association between hypertension and PSCI through its relationship with typical MRI markers of CSVD. These results underscore the importance of early intervention in PSCI and contribute significantly to the diagnosis, treatment, and prevention of the condition. Finally, based on the results of keyword clustering, we systematically and comprehensively discuss the risk factors, pathogenesis, neuropsychological assessment, treatment, and prevention of PSCI. This facilitates a quick understanding of the development process in this field for researchers, provides a scientific basis for clinical practice regarding PSCI, and promotes the overall advancement of the PSCI field.

## 5 Conclusion

The field of PSCI has broad prospects. We identified leading countries, institutions, and leading scholars in the field and analyzed journals and representative literature. We highlighted key research areas in PSCI, such as risk factors, pathogenesis, assessment and diagnosis, treatment and rehabilitation. Moreover, hypertension, WMH, and CMBs play an important role in the correlation between CSVD and PSCI, providing a strong scientific foundation for the prevention and treatment of PSCI. Future research is likely to focus more on areas such as functional connectivity, tool, systematic review, meta-analysis, and CAA, indicating potential new directions for investigation.

## Data Availability

The original contributions presented in the study are included in the article/Supplementary material, further inquiries can be directed to the corresponding authors.

## Footnotes

^1^ https://donghaoren.org/charticulator/docs/getting-started.html

^2^ https://www.mapequation.org/apps/MapGenerator.html
