# Supplementary material for: Knowledge domain and emerging trends in post-stroke cognitive impairment: a bibliometric analysis
Source: Front Aging Neurosci. 2025 Mar 4;17:1525626. doi: 10.3389/fnagi.2025.1525626 (PMC11913868; doi:10.3389/fnagi.2025.1525626)
Supplement: Supplementary file 1 [file Table_1.docx]

***Supplementary Material***

1. **Supplementary Data**

## Data Sources and Search Strategy

The following retrieval strategy was employed: TS=(((Stroke$ OR "Cerebrovascular Accident$" OR "Cerebrovascular Apoplexy" OR Apoplexy OR "Brain Vascular Accident$" OR poststroke OR post-stroke OR "after stroke" OR "Brain Infarct*" OR "Cerebral Infarct*" OR "Subcortical Infarct*" OR "Brain Venous Infarct*" OR "Brain Isch$emia" OR "Isch$emic Encephalopath*" OR "Cerebral Isch$emia” " OR "Intracranial H*morrhage$" OR "Brain H*morrhage$" OR "Cerebral H*morrhage$" OR "Subarachnoid H*morrhage$" OR "Parenchymal H*morrhage" OR "Cerebral H*matoma" OR "Intracranial H*matoma") AND ("Cognitive Dysfunction$" OR "Cognitive Impairment$" OR "Cognitive Disorder$" OR "Cognitive Decline$" OR "Mental Deterioration" OR Dementia OR Amentia)) OR ("post-stroke cognitive impairment" OR "cognitive impairment after stroke" OR "post-stroke cognitive impairment no dementia" OR "post-stroke dementia" OR "PSCIND" OR "PSCI-ND" OR "PSCI" OR "PSD")). In this context, the symbol "$" denotes 0 or 1 character, while "*" represents any character group, including empty characters.

## Data Cleaning

Extracting the original data from the SCI-E database of the WOSCC inevitably leads to duplicate entries. Therefore, we merged records belonging to the same author with different names, such as Mok, Vincent CT and Mok, Vincent; Aamodt, Eva Birgitte and Aamodt, Eva B, among others. Secondly, we combined synonymous entries for countries/regions, such as Taiwan with China, and England with Scotland, Wales, and Northern Ireland, which were grouped as the UK. Finally, we merged keywords with the same meaning. The important keywords were consolidated as follows: "acute stroke," "ischemic stroke," "ischaemic stroke," and "ischemic-stroke" were all merged into "stroke"; and "cognitive decline," "cognitive deficits," "cognitive disorders," and "cognitive impairment" were combined into "cognitive dysfunction."; "poststroke cognitive impairment" and "PSCI" were merged into "post-stroke cognitive impairment"; and "brain infarcts," "cerebral infarction," and "brain infarction" were combined. Additionally, "mini-mental state examination" and "MMSE (Mini-mental State Examination)" were merged, among others. Given that the article primarily focuses on PSCI, "stroke," "cognitive dysfunction," and "post-stroke cognitive impairment" were excluded from the keyword analysis, along with other unrelated terms such as "cells," "participation," "blood," and "disease," among others. After that, the cleaned data were imported into the visualization software applications.

1. **Supplementary Tables**

**TABLE S1 Top 10 countries in the field of PSCI**

| Rank | Country | Documents | Citations | Average Citations | Centrality | Links |
| --- | --- | --- | --- | --- | --- | --- |
| 1 | China | 731 | 8872 | 12.14 | 0.01 | 29 |
| 2 | USA | 305 | 7783 | 25.52 | 0.25 | 42 |
| 3 | UK | 198 | 6445 | 32.55 | 0.28 | 45 |
| 4 | Canada | 109 | 3716 | 34.09 | 0.17 | 38 |
| 5 | Australia | 99 | 2710 | 27.37 | 0.1 | 36 |
| 6 | South Korea | 94 | 1680 | 17.87 | 0.02 | 27 |
| 7 | Netherlands | 91 | 2570 | 28.24 | 0.05 | 30 |
| 8 | Japan | 90 | 1563 | 17.37 | 0.07 | 10 |
| 9 | France | 76 | 2188 | 28.79 | 0.21 | 33 |
| 10 | Germany | 72 | 1980 | 27.50 | 0.1 | 35 |

**TABLE S2 The top 10 journals in the field of PSCI**

| Rank | Journal | Documents | Citations | Average Citations | JCR | IF (2022) |
| --- | --- | --- | --- | --- | --- | --- |
| 1 | Stroke | 94 | 4391 | 46.71 | Q1 | 8.4 |
| 2 | Journal of Stroke & Cerebrovascular Diseases | 74 | 957 | 12.93 | Q3 | 2.5 |
| 3 | Frontiers in Neurology | 63 | 342 | 5.43 | Q2 | 3.4 |
| 4 | Journal of The Neurological Sciences | 37 | 1005 | 27.16 | Q1 | 4.4 |
| 5 | Plos One | 34 | 847 | 24.91 | Q1 | 3.7 |
| 6 | Frontiers in Aging Neuroscience | 33 | 195 | 5.9091 | Q2 | 4.8 |
| 7 | Journal of Alzheimers Disease | 32 | 539 | 16.84 | Q2 | 4 |
| 8 | International Journal of Stroke | 29 | 914 | 31.52 | Q1 | 6.7 |
| 9 | Neurology | 27 | 1025 | 37.96 | Q1 | 10.1 |
| 10 | European Journal of Neurology | 25 | 733 | 29.32 | Q1 | 5.1 |

**TABLE S3 The top 10 journals with the highest citation frequency in the PSCI field**

| Rank | Journal | Documents | Citations | Average Citations | JCR | IF (2022) |
| --- | --- | --- | --- | --- | --- | --- |
| 1 | Stroke | 94 | 4391 | 46.71 | Q1 | 8.4 |
| 2 | Neurology | 27 | 1025 | 37.96 | Q1 | 10.1 |
| 3 | Journal of The Neurological Sciences | 37 | 1005 | 27.16 | Q1 | 4.4. |

**CONTINUED TABLE S3**

| Rank | Journal | Documents | Citations | Average Citations | JCR | IF (2022) |
| --- | --- | --- | --- | --- | --- | --- |
| 4 | Journal of Stroke & Cerebrovascular Diseases | 74 | 957 | 12.93 | Q3 | 2.5 |
| 5 | International Journal of Stroke | 29 | 914 | 31.52 | Q1 | 6.7 |
| 6 | Plos One | 34 | 847 | 24.91 | Q1 | 3.7 |
| 7 | European Journal of Neurology | 25 | 733 | 29.32 | Q1 | 5.1 |
| 8 | Brain | 13 | 639 | 49.15 | Q1 | 15.3 |
| 9 | Journal of Neurology Neurosurgery and Psychiatry | 15 | 634 | 42.27 | Q1 | 11.1 |
| 10 | Archives of Physical Medicine and Rehabilitation | 10 | 606 | 60.6 | Q1 | 4.3 |

**TABLE S4 The main** **citing journals and cited journals in the dual-map overlay of journals related to PSCI research**

| Citing region | Cited region | Z-score |
| --- | --- | --- |
| Molecular,Blology,Immunology | Molecular,Blology,Genetics | 6.165 |
| Neurology,Sports,Ophthalmology | Molecular,Blology,Genetics | 3.423 |
| Neurology,Sports,Ophthalmology | Psychology,Education,Social | 3.188 |
| Molecular,Blology,Immunology | Psychology,Education,Social | 1.865 |

**TABLE S5 The top 10 keywords in the field of PSCI**

| Rank | Kewords | Occurrences | Average citations |
| --- | --- | --- | --- |
| 1 | dementia | 588 | 20.58 |
| 2 | alzheimer's disease | 271 | 25.64 |
| 3 | brain ischemia | 248 | 21.84 |
| 4 | cognition | 246 | 18.75 |
| 5 | memory | 229 | 18.56 |
| 6 | rehabilitation | 208 | 18.75 |
| 7 | risk factor | 201 | 25.09 |
| 8 | recovery | 186 | 18.83 |
| 9 | MMSE | 182 | 26.24 |
| 10 | risk | 170 | 12.94 |

**TABLE S6 The top 10 keywords related to the mechanism in the field of PSCI**

| Rank | Keword | Occurrences | Average citations | Links | Total link strength |
| --- | --- | --- | --- | --- | --- |
| 1 | brain ischemia | 248 | 21.84 | 110 | 1144 |
| 2 | memory | 229 | 18.56 | 150 | 1008 |
| 3 | model | 134 | 21.65 | 103 | 673 |
| 4 | hippocampal | 122 | 18.84 | 110 | 635 |
| 5 | inflammation | 117 | 16.03 | 104 | 524 |
| 6 | protect | 104 | 20.74 | 82 | 474 |
| 7 | oxidative stress | 88 | 22.90 | 87 | 451 |
| 8 | mouse | 81 | 23.41 | 80 | 395 |
| 9 | injury | 81 | 15.28 | 99 | 355 |
| 10 | plasticity | 80 | 16.85 | 108 | 433 |

**TABLE S7 Keywords with long duration in the alluvial flow graph**

| Rank | Keywords | Year | Time of duration |
| --- | --- | --- | --- |
| 1 | synaptic plasticity | 2010 | 13 |
| 2 | oxidative stress | 2010 | 13 |
| 3 | statin | 2010 | 13 |
| 4 | frequency | 2012 | 12 |
| 5 | mortality | 2010 | 11 |
